# Supplementary material for: Understanding the impact of mobility on Plasmodium spp. carriage in an Amazon cross-border area with low transmission rate
Source: PLOS Glob Public Health. 2024 Feb 13;4(2):e0002706. doi: 10.1371/journal.pgph.0002706 (PMC10863871; doi:10.1371/journal.pgph.0002706)
Supplement: S1 Table — (DOCX) [file pgph.0002706.s001.docx]

|  | **SGO Neighbourhood** | | | | | | | | | |
| --- | --- | --- | --- | --- | --- | --- | --- | --- | --- | --- |
| **Characteristic** | **Trois Palet.** | **Village Martin** | **Blondin** | **Esp 1**  **Esp 2**  **Philogène** | **Savane** | **Crique Onozo** | **Village Bambou** | **Lot. Maripa** | **Gabin** | **Adimo** |
| **Total** | 170 | 26 | 45 | 224 | 301 | 179 | 34 | 40 | 86 | 87 |
| **Socio-demographic variables** | | | | | | | | | | |
| **Sex - Male** | 102 (60%) | 11 (42%) | 22 (49%) | 103 (46%) | 126 (42%) | 72 (40%) | 16 (47%) | 18 (45%) | 32 (37%) | 39 (45%) |
| **Age class** |  |  |  |  |  |  |  |  |  |  |
| **Children (<18y)** | 100 (58%) | 15 (58%) | 23 (51%) | 100 (45%) | 151 (50%) | 76 (42%) | 16 (47%) | 20 (50%) | 43 (50%) | 45 (52%) |
| **Adult (≥18y)** | 70 (59%) | 11 (42%) | 22 (49%) | 124 (55%) | 150 (50%) | 103 (58%) | 18 (53%) | 20 (50%) | 43 (50%) | 42 (48%) |
| **Nationality** |  |  |  |  |  |  |  |  |  |  |
| **French** | 131 (77%) | 23 (88%) | 17 (38%) | 139 (62%) | 120 (40%) | 73 (41%) | 21 (62%) | 25 (62%) | 63 (73%) | 59 (68%) |
| **Brazil** | 39 (23%) | 3 (12%) | 27 (60%) | 83 (37%) | 178 (59%) | 105 (59%) | 13 (38%) | 15 (38%) | 23 (27%) | 28 (32%) |
| **Other** | 0 (0%) | 0 (0%) | 1 (2%) | 2 (1%) | 2 (1%) | 1 (1%) | 0 (0%) | 0 (0%) | 0 (0%) | 0 (0%) |
| **Ethnic group** |  |  |  |  |  |  |  |  |  |  |
| **Amerindian** | 151 (89%) | 25 (96%) | 31 (69%) | 209 (93%) | 161 (53%) | 64 (36%) | 19 (56%) | 30 (75%) | 46 (53%) | 76 (87%) |
| **Other** | 19 (11%) | 1 (4%) | 14 (31%) | 15 (7%) | 140 (47%) | 115 (64%) | 15 (44%) | 10 (25%) | 40 (47%) | 11 (13%) |
| **Risk factors** | | | | | | | | | | |
| **Slash-and-burn farming** | 130 (76%) | 8 (31%) | 35 (78%) | 166 (74%) | 124 (41%) | 45 (25%) | 8 (24%) | 13 (32%) | 24 (28%) | 39 (45%) |
| **Hunting** | 47 (28%) | 3 (12%) | 10 (22%) | 39 (17%) | 40 (13%) | 23 (13%) | 5 (15%) | 4 (10%) | 9 (10%) | 12 (14%) |
| **Fishing** | 97 (57%) | 12 (46%) | 25 (56%) | 58 (26%) | 64 (21%) | 55 (31%) | 8 (24%) | 5 (12%) | 19 (22%) | 15 (17%) |
| **Visits to gold mining sites** | 12 (7%) | 1 (4%) | 0 (0%) | 4 (2%) | 10 (3%) | 6 (3%) | 1 (3%) | 1 (3%) | 3 (4%) | 2 (2%) |
| **Travel to OIT** | 73 (43%) | 1 (4%) | 7 (16%) | 44 (20%) | 26 (9%) | 25 (14%) | 5 (15%) | 4 (10%) | 3 (4%) | 19 (22%) |
| **Travel to Upper Oyapock river** | 0 (0%) | 0 (0%) | 0 (0%) | 2 (1%) | 2 (1%) | 7 (4%) | 0 (0%) | 0 (0%) | 8 (9%) | 1 (1%) |
| **Travel to Oiapoque** | 1 (1%) | 0 (0%) | 1 (2%) | 6 (3%) | 27 (9%) | 41 (23%) | 3 (9%) | 1 (3%) | 4 (5%) | 4 (5%) |
| **Travel to Regina** | 2 (1%) | 0 (0%) | 1 (2%) | 5 (2%) | 2 (1%) | 4 (2%) | 0 (0%) | 0 (0%) | 2 (2%) | 2 (2%) |
