# Supplementary material for: Understanding the impact of mobility on Plasmodium spp. carriage in an Amazon cross-border area with low transmission rate
Source: PLOS Glob Public Health. 2024 Feb 13;4(2):e0002706. doi: 10.1371/journal.pgph.0002706 (PMC10863871; doi:10.1371/journal.pgph.0002706)
Supplement: S2 Table — (DOCX) [file pgph.0002706.s002.docx]

|  | **2017** | | | **2018** | | |
| --- | --- | --- | --- | --- | --- | --- |
| **Characteristic** | **No *plasmodium* carriage** | ***Plasmodium* carriage** | **p-value^1^** | **No *plasmodium* carriage** | ***Plasmodium* carriage** | **p-value^1^** |
| **Total** | 1103 | 89 |  | 1157 | 35 |  |
| **Sex - Male** | 499 (45%) | 42 (47%) | 0.7 | 523 (45%) | 18 (51%) | 0.5 |
| **Age (mean)** | 17 (8, 35) | 23 (11,37) | 0.13 | 17 (8, 35) | 24 (17, 35) | 0.044 |
| **Ethnic group** |  |  | 0.2 |  |  | 0.13 |
| **Amerindian** | 746 (68%) | 66 (74%) |  | 784 (68%) | 28 (80%) |  |
| **Other** | 357 (32%) | 23 (26%) |  | 373 (32%) | 7 (20%) |  |
| **Vector density around participant’s home** |  |  | <0.001 |  |  | <0.001 |
| **Low** | 680 (62%) | 23 (26%) |  | 693 (60%) | 10 (29%) |  |
| **Medium** | 156 (14%) | 5 (6%) |  | 157 (14%) | 4 (11%) |  |
| **High** | 267 (24%) | 61 (69%) |  | 307 (27%) | 21 (60%) |  |
| **Slash-and-burn farming** | 528 (48%) | 64 (72%) | <0.001 | 565 (49%) | 27 (77%) | <0.001 |
| **Hunting** | 168 (15%) | 24 (27%) | 0.004 | 183 (16%) | 9 (26%) | 0.12 |
| **Fishing** | 314 (28%) | 44 (49%) | <0.001 | 340 (29%) | 18 (51%) | 0.005 |
| **Visits to gold mining sites** | 33 (3%) | 7 (8%) | 0.025 | 37 (3%) | 3 (9%) | 0.11 |
| **Travel to OIT** | 176 (16%) | 31 (35%) | <0.001 | 191 (17%) | 16 (46%) | <0.001 |
| **Travel to Oiapoque** | 81 (7%) | 7 (8%) | 0.9 | 86 (7%) | 2 (6%) | >0.9 |
| **Travel to Upper Oyapock river** | 20 (2%) | 0 (0%) | 0.4 | 20 (2%) | 0 (0%) | >0.9 |
| **Travel to Regina** | 15 (1%) | 3 (3%) | 0.15 | 17 (2%) | 1 (3%) | 0.4 |

^1^Pearson's Chi-squared test; Wilcoxon rank sum test; Fisher's exact test
