# Supplementary material for: Understanding the impact of mobility on Plasmodium spp. carriage in an Amazon cross-border area with low transmission rate
Source: PLOS Glob Public Health. 2024 Feb 13;4(2):e0002706. doi: 10.1371/journal.pgph.0002706 (PMC10863871; doi:10.1371/journal.pgph.0002706)
Supplement: Attachment — Submitted filename: Response to rewievers.docx [file pgph.0002706.s003.docx]

Response to Reviewers

PGPH-D-23-01522

« The impact of mobility on Plasmodium spp. carriage in border areas with a low malaria transmission rate: an example in Amazonia »

Dear Dr André Machado, dear reviewers,

Thank you for sending us the reviews of our manuscript entitled “The impact of mobility on Plasmodium spp. carriage in border areas with a low malaria transmission rate: an example in Amazonia ”. We are very grateful to the reviewers and editors for their helpful and constructive comments. We have modified the manuscript in response to the comments and believe it has been substantially strengthened.

We would like to mention that those results have never been submitted elsewhere.

Due to reasons beyond our control and if it possible, we would need Jean Gaudart (jean.gaudart@univ-amu.fr) to be the corresponding author for further communications.

We thank you for your expert editorial consideration of this revised manuscript and hope you will find this revision suitable for publication in PLOS Global Public Health.

Yours sincerely,

*Helene Trehard*

*The corresponding author on behalf of all authors*

**Potential Copyright Issues**

"Figure 1-3: please (a) provide a direct link to the base layer of the map (i.e., the country or region border shape) and ensure this is also included in the figure legend; and (b) provide a link to the terms of use / license information for the base layer image or shapefile. We cannot publish proprietary or copyrighted maps (e.g. Google Maps, Mapquest) and the terms of use for your map base layer must be compatible with our CC-BY 4.0 license.

Note: if you created the map in a software program like R or ArcGIS, please locate and indicate the source of the basemap shapefile onto which data has been plotted.

If your map was obtained from a copyrighted source please amend the figure so that the base map used is from an openly available source. Alternatively, please provide explicit written permission from the copyright holder granting you the right to publish the material under our CC-BY 4.0 license.

Please note that the following CC BY licenses are compatible with PLOS license: CC BY 4.0, CC BY 2.0 and CC BY 3.0, meanwhile such licenses as CC BY-ND 3.0 and others are not compatible due to additional restrictions.

Response: Dear Editor, thank you very much for your reminder. The map backgrounds used are sourced from OpenStreetMap, as indicated in the manuscript and on the Figures. In compliance with OpenStreetMap's requirements, we have added the disclaimer " © OpenStreetMap contributors" on the maps and acknowledged them in the acknowledgments section (Map data use OpenStreetMap® contributors. OpenStreetMap® is *open data*, licensed under the Open Data Commons Open Database License (ODbL), available at <https://www.openstreetmap.org/copyright>.). Would you like the links to be written out in full in the legend or the tittle of each figure?

**Data Availability Statement**

Have the authors made all data underlying the findings in their manuscript fully available (please refer to the Data Availability Statement at the start of the manuscript PDF file)? NO

The PLOS Data policy requires authors to make all data underlying the findings described in their manuscript fully available without restriction, with rare exception. The data should be provided as part of the manuscript or its supporting information, or deposited to a public repository. For example, in addition to summary statistics, the data points behind means, medians and variance measures should be available. If there are restrictions on publicly sharing data—e.g. participant privacy or use of data from a third party—those must be specified.

Response: Thank you for this suggestion. We added some information to the Data Availability Statement at the start of the manuscript PDF file.

Manuscript modification (Data Availability Statement): The datasets generated and analysed during the current study are not publicly available as specific French National Data Protection Commission ("Commission Nationale de l'Informatique et des Libertés" or CNIL) authorization is required for the transfer of personal data, in order to protect participant privacy. The datasets may be obtained from the corresponding author upon reasonable request only with specific authorization from the CNIL (for CNIL authorization contact dpo@univ-amu.fr).

**Point-by-point response to Reviewer 1**

Reviewer #1: A well planned, structured study with a clear and key research question or problem: Cross-border malaria and the elimination route in the region. The discussion of the results is pertinent as well as the recommendations derived from the results.

Response: Thank you for reviewing our manuscript and for your comment.

**Point-by-point response to Reviewer 2**

Reviewer #2: The authors have presented a study holds significant importance in the context of malaria elimination in cross-border areas. It highlights the challenges faced in achieving and maintaining malaria elimination, particularly in regions like Amazonia. The study reveals that despite a substantial reduction in malaria incidence over the past decade, the final steps toward elimination remain the most difficult, especially in international border regions. Key findings of the study underscore the crucial role of mobility in Plasmodium spp. carriage. It identifies factors such as travel to adjacent Indigenous territories, vector density around participants’ homes, slash-and-burn farming, and age as contributing to malaria transmission tailored to different types of mobility. Additionally, the study’s implications are significant for public health strategies, suggesting that strategies like reactive case detection and treatment in areas frequently visited by mobile populations are essential in cross-border regions. By recognizing the impact of mobility on malaria transmission and tailoring interventions accordingly, this reach contributes to the ongoing efforts to eliminate malaria, particularly in challenging border areas.

Response: First of all, thank you for reviewing our manuscript. We appreciate all the comments and questions about our work. We hope that their responses below will bring more clarity.

**Point 1**

However, the study has some limitations, such as data from only two years and entomology data was estimative. Thus, a principal suggestion is changing the title for “Understanding the Impact of Mobility on Malaria Transmission in an Amazon Cross-Border Area with Low Transmission Rates”, because the real “impact” was not found and describe due data limitation, if you consider that you found the impact, describe in the abstract and in the conclusion section.

Response: Thank you for that suggestion. We chose to follow your advice as we can’t assess the impact of mobility with our data. We kept “*Plasmodium* spp. carriage” instead of “Malaria transmission” as we prefer to be more specific in the tittle and discuss the link between *Plasmodium* carriage and malaria transmission in the discussion.

Manuscript modification (line 4-7): Understanding the impact of mobility on *Plasmodium* spp. carriage in an Amazon cross-border area with low transmission rate

**Point 2**

If you choose to retain the title, please note that “spp.” should not be italicized. The same observation applies to short title.

Response: Thank you for this remark. We have taken these into account by adding corrections in track changes.

**Point 3**

Line 38: spp. should not be italicized. Please, review the entire manuscript for this formatting.

Response: Thank you for this remark. We have taken these into account by adding corrections in track changes.

**Point 4**

Line 44: Why were patients treated only if it was possible?

Response: Thank you for this remark. As we mentioned, the patients were treated only if positive (PCR). Patients with *P. vivax* infection get treated by chloroquine for 3 days followed by 14 days of primaquine (0.5mg/kg) in the absence of medical contraindications (pregnancy, G6PD deficiency or breastfeeding an infant below the age of 6 months).

**Point 5**

Line 66: P. falciparum is first time mentioned in the text, so you have to describe it without using the abbreviation.

Response: Thank you for this remark. We described it without using the abbreviation for this first time, and did the same for *P. vivax*.

Manuscript modification (line 67 and 82): (defined as annual parasite incidence of < 10000 cases per 100000 population and a Plasmodium falciparum rate <1%).

Plasmodium vivax accounts for 97% of malaria cases in French Guiana.

**Point 6**

Line 114: Materials AND methods

Response: Thank you for this remark. We have taken this into account.

Manuscript modification (line 115): Materials and methods

**Point 7**

Fig. 1: the quality of the figure is not good.

**Response:** Thank you for this remark, we have improved the quality.

**Point 8**

Fig 2: The legend of the figure is difficult to read and does not clearly identify each color.

**Response:** Thank you for this remark, we have removed the colors that were not necessary for understanding the article.

**Point 9**

Line 189: P. vivax at 0.5 parasite/ μL?

Response: Thank you for this remark. We have changed p/μL by parasite/μL.

Manuscript modification (line 191): with a sensitivity of 1 parasite/μL for all the species except P. vivax at 0.5 parasite/μL.

**Point 10**

Line 205: Legend for Fig 3: check the scientific name of Anopheles darlingi.

**Response:** *Anopheles darlingi* is described as the primary malaria vector in French Guiana according to the early entomological studies (1,2). The scientific name of the mosquito has been italicized in the figure title.

1.	Adde A, Dusfour I, Vezenegho SB, Carinci R, Issaly J, Gaborit P, et al. Spatial and Seasonal Dynamics of Anopheles Mosquitoes in Saint-Georges de l’Oyapock, French Guiana: Influence of Environmental Factors. J Med Entomol. 1 mai 2017;54(3):597‑605.

2.	Girod R, Roux E, Berger F, Stefani A, GAborit P, Carinci R, et al. Unravelling the relationships between Anopheles darlingi (Diptera: Culicidae) densities, environmental factors and malaria incidence: understanding the variable patterns of malarial transmission in French Guiana (South America). Ann Trop Med Parasitol. mars 2011;105(2):107‑22.

**Point 11**

Fig 4. Check the font type used in some of text box.

**Response:** Thank you for this remark. The font type has been harmonized.

**Point 12**

Lines 252 to 257 and Fig. 4: Why you had only 1,192 participants who took part in both surveys have available PCR results for both 2017 and 2018 when you had 1,501 with PCR result in 2017 and 1,232 with PCR results in 2018, and you had only 290 lost follow-ups?

Response: Thank you for your comment that allow to clarify this point. This number is explained by the fact that PCR tests were not usable for all participants at both times of the study. At T1, 1,501 of the 1,567 participants had a usable PCR test, and only 1,232 of the 1,277 remaining at T2. Some of the T1 participants who had a usable PCR test at T1 were not lost to follow-up, but neither did they have a usable PCR test at T2 (n=19). Similarly, some of the T2 participants who had a usable PCR test at T2 did not have a usable PCR test at T1 (n=40).

The 1192 people with a usable PCR test at both T1 and T2 therefore corresponds to the 1501 participants with a usable PCR test at T1 minus those who did not have a usable test at T2 minus those lost to follow-up. Or 1232 participants with a usable PCR test at T2 minus the 40 participants without a usable test at T1.

**Point 13**

Line 261 to 263 and Table 01: is this information only form 2017? If not, why did you only describe the median age from 2017?

Response: Thank you for your comment that allow to clarify this point. For this study, we were able to collect dates of birth, but age seemed more interesting to use in the paper to allow comparison with other data. As the study took place over two years, we arbitrarily chose to describe the age of the participants on December 31, 2017, i.e. approximately at the end of time 1 of the study. We could have chosen the age at study entry, but as the inclusion period lasted 3 months, it seemed more relevant to us to set a common date to get a "snapshot" of the population age distribution in our study area.

**Point 14**

Table 1: why did you not include data for female in the Table?

Response: Thank you for your comment that allow to clarify this point. There was no missing data for this variable, so we decided to present only one of the two modalities of the variable to avoid overloading the table. We chose instead to present the male modality, as they have been found to be at greater risk of malaria in previous studies in the area.

**Point 15**

Line 272: I suggest writing (Table 1 and S1 Table) instead of (S1 Table) (Table 1).

Response: Thank you for this suggestion, which makes the text more readable. We changed it in the manuscript.

**Point 16**

Table 2: I suggest providing information about Regina in the setting section, as you did for Oiapoque.

Response: Thanks for spotting this oversight, we've added it in the setting section of the manuscript.

Manuscript modification (line 132-133): Apart from SGO and Oiapoque, the Oyapock river basin consists of remote villages scattered along both sides of the river, one village on the way to Cayenne (Regina) and Oiapoque Indigenous Territories (OIT) located along the lower Oyapock river on the Brazilian side, which extend 100 km to the southeast (Fig 1).

**Point 17**

Lines 296 to 300: Is this information about 2017 and 2018? If so, why did you describe the percentage of travel to high-risk areas only for 2018?

Response: Thank you for spotting this mistake. The data regarding travel to high-risk areas were collected both in 2017 and 2018 for travels to indigenous territories of Oyapock and in 2017 for other destinations. Since travels to this area are mainly cultural and family-oriented, we assumed that residents who travelled to this area at least once in a year did so at least annually. We have acknowledged this limitation in our discussion. Further clarifications have been added to the methods, results and discussion (limitations) section of the article.

Manuscript modification (line 219-220): The data regarding the three first areas were collected only during the first survey while the data regarding the last area were collected both in 2017 and 2018.

Manuscript modification (line 301): In terms of travel to high-risk areas, 17% went to the OIT in 2017 and/or 2018 (n=207),

Manuscript modification (line 425-426): Moreover, some data regarding trips in high risk areas were collected only in one survey.

**Point 18**

Fig 5. The font used in the Legend is not easy to read, the quality of the figure is not good, and the circle indicating the number of cases of Plasmodium is unclear.

**Response:** Thank you for your feedback, we have clarified the legend and adjusted the circles indicating the number of cases to make the figure more understandable.

**Point 19**

Line 324: Legend for Figure 6: Plasmodium should be written with a lowercase “p”.

Response: Thank you for this suggestion. We added “spp.” where it was missing and changed the “plasmodium” in “Plasmodium”. We also added “spp.” when it was missing.

Manuscript modification (line 327): The solid black line represents the *Plasmodium* spp. carriage/no *Plasmodium* spp. carriage ratio as a function of age.

**Point 20**

Line 342: Abbreviate the genus Plasmodium for the specie P. vivax.

Response: Thank you for this suggestion. We changed it in the manuscript.

**Point 21**

Line 348 and 428: Abbreviate the genus Anopheles darlingi for the specie An. darlingi.

Response: Thank you for this suggestion. We changed it in the manuscript.

**Point 22**

Fig 7. The quality is not good, and the text is not visible.

Response: Thank you for your comment, we have enlarged the text to make it easier to read and improved the quality of the figure.

**Point 23**

Line 387: This is a common practice in malaria-free countries. – Add the reference.

Response: Thank you for this suggestion. We added the following reference: [International travel and health, WHO](https://www.who.int/publications/i/item/9789241580472)
